# Estimation of Cadmium in Muscles of Five Freshwater Fish Species from Manzalah Lake, and Possible Human Risk Assessment of Fish Consumption (Egypt)

**DOI:** 10.1007/s12011-022-03188-5

**Published:** 2022-03-24

**Authors:** Heba H. Abdel-Kader, Mohamed H. Mourad

**Affiliations:** National Institute of Oceanography and Fisheries (NIOF), Alexandria, Egypt

**Keywords:** Cadmium, Bioaccumulation, Risk assessment, Target hazard quotient, Target carcinogenic risk, Manzalah Lake, Egypt

## Abstract

The Egyptian government devised a plan in 2016 to improve the unique ecological significance of northern lakes, which mentioned Manzalah Lake in the Egypt Vision 2030. In this regard, this study investigated cadmium (Cd) content in five freshwater fish species collected from Manzalah Lake in Egypt at 2018 by local fishermen. According to the findings, *Clarias gariepinus* recorded the highest concentration of Cd (1.40 ± 0.2 μg/g) and the lowest concentration was recorded in *O. aureus* (1.19 ± 0.2 μg/g). Cadmium contents of all species were largely above the permissible level of the Food Agricultural Organization (FAO)/World Health Organization (WHO) and Commission Regulation (EC). The estimated daily intake (EDI), the estimated weekly intake (EWI), and the percentages of provisional tolerable weekly intake (PTWI %) values for Cd in the *C. gariepinus* > *Sarotherodon galilaeus* > *Tilapia zillii* > *Oreochromis niloticus* > *Oreochromis aureus* which consumed by children, teenagers, and adults were much higher than the PTWI values established by FAO/WHO. In addition, *C. gariepinus* consumed by children showed the highest value of the target hazard quotient (THQ) (5.83 a day or 40.81 a week) while *O. aureus* that ingested by adults showed the lowest level (1.06 a day or 7.42a week). The target carcinogenic risk (TCR) of *C. gariepinus* in children had the greatest level (2.21 × 10^−3^ a day or 1.55 × 10^−2^ a week), whereas *O. aureus* in adults had the lowest level of TCR (4 × 10^−4^ a day or 2.81 × 10^−3^ a week). THQs values of Cd in the five studied species were found higher than one. Moreover, TCRs values of Cd in the five species were exceeded the US Environmental Protection Agency guideline USEPA permissible limits suggesting that a daily or weekly consumption of these species could lead to a high risk non-carcinogenic and carcinogenic for humans.

## Introduction

Aquatic pollution is a very important global environmental concern due to the accumulation of heavy metals resulting from industrial activities or rock erosion, and the consumption of aquatic species contaminated with these heavy metals caught from these environments may threaten the health of consumers [1–4]. Cadmium (Cd) is a hazardous heavy metal with a specific gravity of 8.65 times that of water [5]. It occurs naturally in small concentrations in the environment [6]; the aquatic ecosystem is polluted by Cd due to soil erosion caused by rain through natural pollution and man-made contamination (90%) due to liquid flows from various industrial, urban, and agriculture activities [6–11]. As a significant chemical contaminant of the watery environment, Cd poses a substantial hazard to aquatic species, particularly fish [8]. Exposure to Cd in the environment may result in the absorption of significant amounts of the element, contaminating the organism [9]. Fish uptake Cd through two main mechanisms are as follows: directly from water through its free ionic state Cd (II) gills adsorbed or organically consumed elements through the intestine; and indirectly by entering the chloride cells in gills through via calcium channels. Once within the cells, the metal becomes available for interaction with cytoplasmic components constituents such as enzymes (producing negative impacts) and metallothioneine (probably causing detoxification) [7, 11, 12].

Cadmium is extremely harmful due to its extremely potential toxicity even at low levels, its persistence in the environment, and its proclivity for bioaccumulation in aquatic biota. In the aquatic biota, Cd is not digested by the body and it accumulates in the soft tissues and becomes poisonous, and as a direct consequence of their bioaccumulation, the food chain has become contaminated, affecting the entire ecological activity. Nowadays, global attention becomes more critical for African countries such as Egypt, where the pressure from exploding the population requires a lot of food supply [7, 13–15].

Healthy fish is considered a nutritious food that provides omega-3 polyunsaturated fatty acids, proteins, vitamins, and a wide range of other important nutrients [16, 17]. Plus, many researchers recorded that fish reduced the risk of heart disease and normal neurodevelopment in children [18]. So, it should be eaten 2–3 times per week [19]. On the contrary, if a toxic substance such as Cd was accumulated in fish tissue, it becomes a health risk [20]. Thus, as a bio-indicator, fish can be used to monitor trace element residues in aquatic settings [1, 2, 4, 16, 20–22]. Many researchers estimated Cd in hazardous concentrations in a variety of aquatic environments [4, 7, 13–16, 21, 22].

Since fish occupy the higher trophic level in aquatic environments, there is a larger possibility of Cd transfer to living organisms, including humans, especially for fish consumers all days of the week [1, 2, 16, 20–22]. The symptoms were documented as signs of acute Cd poisoning in humans such as nausea, vomiting, stomach aches, and trouble breathing that were all documented as signs of acute Cd poisoning in humans [23]. While the chronic Cd exposure symptoms were respiratory disease, kidney failure, weak bones, eczema, anemia, osteoarthritis, cognitive difficulties, headaches, growth impairment, asthma, and cardiac diseases [23]. Moreover, Cadmium may induce the destruction of testicular plus erythrocyte tissues [24], and a higher risk of lung cancer [25, 26].

The major goal of this study was to quantify the levels of Cd in four different freshwater species of tilapia, namely, Nile tilapia *Oreochromis niloticus*, Blue *tilapia Oreochromis* aureus, Mango tilapia *Sarotherodon galilaeus*, and Redbelly tilapia *Tilapia zillii*, as well as African sharptooth catfish *Clarias gariepinus* and compare results to those of other studies as well as the European Commission’s regulatory limit levels. Local fishermen caught these different species from different sites at Manzala Lake, Egypt.

Evaluation of human risk for five species has been estimated using the estimated daily or weekly intake (EDI-EWI), maximum daily or weekly intake (MDI-MWI), the provisional tolerable weekly intakes (PTWI %), target hazard quotients (THQs), and target carcinogenic targets (TCRs) for Cd consumed by a child 15 kg, teenager 40 kg, and an adult 70 kg.

## Materials and Methods

### Samples for Testing

In this research, approximately 75 freshwater specimens of *O. niloticus*, *O. aureus*, *S. galilaeus*, *and T. zillii*, four tilapia species, and catfish *C. gariepineus* were caught at random in May 2018 from different sites along Manzalah Lake, Egypt, by local fishermen while they were fishing at the Lake. These fish species were selected based on their distribution across the lake as well as frequent consumption by the consumers, and then brought alive to the National Institute of Oceanography and Fisheries (NIOF), Egypt, physiological Lab for analyses.

### Study Methods

During the night, all glass wares were immersed in 10% (v/v) nitric acid before washing in 10% (v/v) hydrochloric acid, then washed by double distilled water then dried until use. All chemicals used were Merck, Germany; analytical reagents of the highest quality. The element standard solution used for the calibration curve was produced by diluting stock solutions of 1000 mg/l (Merck-Germany). Each fish was dissected and the skin was removed and then the musculature was collected and wrapped in aluminum foil, separately packaged in a clean polyethylene bag with an identifying number and collection date, fish are then stored frozen at − 20 °C until they sampled and digested. In 25-mL Erlenmeyer flasks, 1 g of fish tissue sample was properly weighed, 5 mL nitric acid (65% from Merck, Germany) to each sample was added, and then samples were placed overnight digesting slowly. Following that, 2.5 mL of perchloric acid (72% from Merck, Germany) was poured into each sample. Digestion was done in a water bath at 150 °C for 6 h or until solutions became clear and close to dryness. After cooling, the solutions were transferred to 50 mL of polyethylene bottles and filled with distilled water to 25 mL. The solution was subsequently filtered into a clean glass beaker with 0.45 μm Whatman filter paper No. 42. By adding more deionized water, the filtrate was diluted to up to 50 mL. The metal measurements were done using a Perkin-Elmer AAnalyst 800 using a graphite furnace atomic absorption spectrophotometry (GF-AAS). Cd concentrations in fish were measured in microgram per gram (μg/g) on a wet weight (ww) basis. Three replicate blank samples were digested using the same technique for Cd contents in the fish samples that were examined. LOD was determined using the standard deviation (SD) of the three replicate blanks for Cd 0.04 μg/g ww. The quality of analyses was monitored using the certified fish muscles references material (DOLT-4 dogfish liver). The concentration of Cd was 0.05 mg/kg − 1 established by the European Union [27] in reference material human diets. The reference material’s replicate analysis revealed high accuracy, with an element recovery rate of 98%.

### Human Risk Assessment Protocol

#### ***Estimated Daily and Weekly Intake of Trace Metals***

The estimated daily or weekly intake (EDI-EWI) of Cd via fish consumption were determined using the next two equations:

$$\text{EDI} = \frac{{\mathrm{C}}\times {\text{IR}}}{\text{BW}}$$

where *C* is the concentration of cadmium in fish samples (μg/g-ww), *IR* is the daily ingestion rate of fish (62.25 g/person/day) according to Ministry of Agriculture and Land Reclamation, Egypt [28], and Central Agency for Public Mobilization and Statistics [29], and *BW* is the average body weight (15 kg a child, 40 kg teenager, and 70 kg an adult) [30, 31].

$$\text{EWI}=\text{EDI}\times {7}$$

EWI was compared to the provisional tolerable weekly intakes (PTWI), which were determined from FAO/WHO food safety standards for Cd. When EWI is smaller than PTWI, it means that food consumption does not pose a significant health risk to consumers [32].

$$\text{PTWI \%} =\frac{\text{EWI}}{{\text{PTWI}}}\times {100}$$

The percentage of PTWI was determined for Cd using the possible safety reference dose recommended by FAO/WHO [32]. The percent of PTWI was calculated for Cd using the FAO/WHO potential safety reference dosage [32].

$$\begin{array}{c}\text{MDI} = \frac{\text{PTWI }\times \text{ BW}}{\mathrm{C}\times  7}\\ \text{MWI} = \text{MDI }\times  7\end{array}$$

Maximum Daily Intake MDI (in grams) was based on the weekly safety intake of fishes that children, teenagers, and adults that should attain PTWI [32].

#### Non-carcinogenic Risk Estimation

Target hazard quotient (THQ) was calculated [33] by the following formula:

$$\text{THQ} = \frac{\text{EF }\times \text{ED }\times \text{IR }\times \mathrm{C}}{{\text{RfD}} \, \times \text{ WAB }\times \text{ ATn}}\times {10}^{-3}$$

where, *EF* is the exposure frequency (days/year); *ED* is the exposure duration (years); *IR* is the ingestion rate (g/day); *C* is the metal concentration in fish (μg/kg); *RfD* is the oral reference dose (Cd = 1.0 × 10^−3^ mg kg − 1 day − 1); *WAB* is the average body weight (kg); and *ATn* is the average exposure time for non-carcinogens (days/year × ED). When THQ ≤ 1, the non-carcinogenic risk is acceptable, but if THQ ≥ 1, the non-carcinogenic risk is considerable [33].

#### Carcinogenic Risk Estimation

Target carcinogenic risk (TCR) was calculated USEPA [33] by the following formula:

$$\text{TCR} = \frac{\text{EF }\times \text{ ED }\times \text{ IR }\times \mathrm{C}\times \text{ CSF}}{\text{WAB }\times \text{ ATc}}\times {10}^{-3}$$

where, *EF* is the exposure frequency (days/year); *ED* is the exposure duration (years); *IR* is the ingestion rate (g/day); *C* is the metal concentration in fish (μg/kg); *CSF* is the cancer slope factor (Cd = 0.38 mg kg − 1 day − 1) [34]; *WAB* is the average body weight (kg); and *ATc* is the average time for carcinogens (days/year × ED). US EPA [35] states that the CR values lower than 1 × 10^−6^ (risk of developing cancer is 1 in 1,000,000 over a lifetime of 70 years) is considered to be negligible, CR values above 1 × 10^−4^ (risk of developing cancer is 1 in 10,000) is considered unacceptable, and CR values between 10^−6^ and 10^−4^ is generally considered as acceptable, CR ≤ 10–3 to 10–1, high risk; and ≥ 10–1, very high risk [36, 37].

### Statistical analysis

Means and standard errors of the concentrations of cadmium in five fish species were recorded; the resulting data were analyzed using one-way analysis of variance to evaluate the differences in the cadmium levels among the various fish species tested. Differences among means were estimated using Tukey honestly significant difference analysis (Turkey’s HSD); meanwhile one-tailed *t*-test was carried out in order to determine the significant difference among cadmium means.

## Results and Discussion

Cadmium is a severely toxic heavy metal that is completely unessential to all organisms. The accumulation of Cd in freshwater bodies such as lakes and rivers has become a major source of concern for environmentalists as industrialization grows [4, 8–10].

**Fig. 1:**
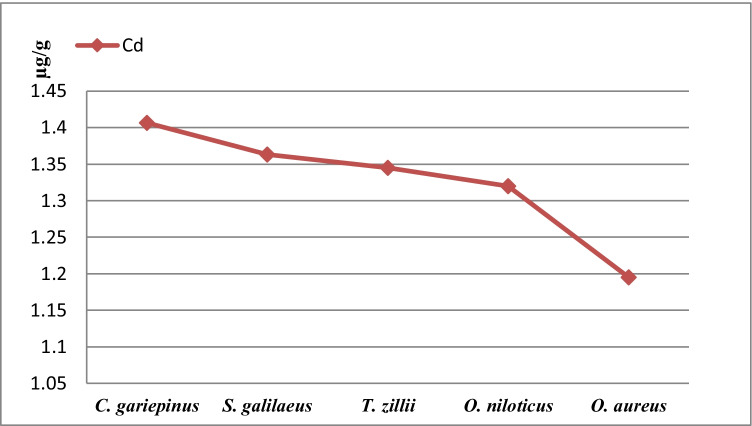
Bioaccumulation of Cd (μg/g wet weight) in five fish species from Manzalah Lake, Egypt

In this study, Table 1 displayed the Cd concentrations in the four tilapia species and catfish *C. gariepinus* from Manzalah Lake, Egypt. The present research manifested that the average concentrations of Cd in five species were insignificantly increased (*p* > 0.05) and recorded as follows: *C. gariepinus* > *S. galilaeus* > *T. zillii* > *O. niloticus* > *O. aureus* (μg/g ww). Figure 1 demonstrated that *C. gariepinus* recorded the highest concentration of Cd level (1.40 ± 0.2 μg/g) and the lowest concentration was detected in *O. aureus* (1.19 ± 0.2 μg/g). In this study, Cd contents of all species were largely above the permissible level of Egyptian Organization for Standardization and Quality Control EOSQC [38] 0.1 μg/g, FAO/WHO [39] 0.05 μg/g, FAO [40] 2.0 μg/g, EPA [41] 0.5–1 μg/g, EC [27] amended by EC [42, 43] 0.050 μg/g, WHO [44] 0.5 μg/g, and FAO [45] 0.1 μg/g. *O. aureus* from Edku Lake, Egypt, recorded the significant highest level of Cd (1.09) > S. *galilaeus* (0.90) > O. *niloticus* (0.81) [46]. Saeed and Shaker [47] recorded the Cd level in muscle of *O. niloticus* collected from Manzala Lake, Egypt, was 10.36 μg/g which was much higher than this research. From the Danube River, Grocka, the mean Cd content in in the muscle of carp was 0.082 μg/g, which was lower than our results [48].

**Table 1:** Mean average cadmium (μg/g wet weight) concentrations in five fish species from Manzalah Lake, Egypt

Fish	Cd concentrations (μg/g wet weight)			
	Mean	± SE	Mix	Max
*C. gariepinus*	1.40	0.15	1.20	1.70
*S. galilaeus*	1.36	0.11	1.20	1.44
*T. zillii*	1.34	0.12	1.11	1.68
*O. niloticus*	1.32	0.09	0.88	1.68
*O. aureus*	1.19	0.04	0.91	1.67

Hamed et al. [2] recorded that the Cd concentrations in *Brachidontes pharaonis* (0.432) > *Holothuria polii* (0.120) > *Pinctada radiate* (0.055) (μg/g wet weight) were collected along Egypt’s Alexandria Coast. The estimated Cd values in marine edible puffer fishes from Gulf of Mannar Marine Biosphere Reserve, South India, varied from 0.01–0.79 mg/kg μg/g, which was substantially lower than our data [21]. Alipour et al. [49] evaluated the Cd levels in the edible muscle tissues of *Rutilus rutilus* from the Miankaleh international wetland, Iran (0.26 mg kg − 1) that was much lower than this research. Jarosz-Krzeminska et al. [50] estimated the Cd content in smoked rainbow trout inhabiting Polish fish farm (60.0 µg/kg) in the muscle of *Tilapia zillii* inhabiting the Elemi River in Ado-Ekiti, Dewunmi et al. [51] observed a higher accumulation of Cd (0.56 mg/kg) from Elemi River, Ado-Ekiti, south west Nigeria which was much lower than this research. Nwabueze [52] found 2.16 mg/kg of Cd in fish collected from the Forcados River; southern Nigeria was much higher than this research.

Sallam et al. [53] reported the Cd levels in Nile tilapia (0.024) > African catfish (0.020) > flathead gray mullet (0.006 μg/g) caught from Lake Manzalah, Egypt. Their findings were attributed to a lack of fresh water supply needed to transport sewage wastes from the lagoon to the sea. Ali et al. [22] showed that the mean levels of Cd varied from 0.014 to 0.022 mg/kg in *P. sophore* from Challawa River, northwestern Nigeria, which was under the permissible limit was much lower than this research. Abdel Kader and Mourad [54] determined the Cd levels in fish samples obtained from Burullus Lake in Egypt as follows: *C. gariepinus* (1.66) > *O. aureus* (1.54) > *T. zillii* (1.49) > *S. galilaeus* (1.46) > *O. niloticus* (1.10 μg/g). Mielcarek et al. [55] estimated that Cd, ranged 0.02–97.0 μg/kg of freshwater fish species from Poland especially smoked fish products. Łuszczek-Trojnar et al. [56] observed that Cd levels in smoked rainbow trout (10.0 ± 1.0 μg/kg) from fish farm, Poland. Llamazares Vegh et al. [57] recorded that Cd levels ranged from (0.01 to 0.04) in juvenile fishes from the Lower Paraná River (Argentina). Mortazavi et al. [58] estimated that Cd in the muscles of rainbow trout was 0.123 mg/kg from marketed Khorramabad City, Iran. Lidwin-Każmierkiewicz et al. [59] estimated that Cd levels in pike, bream, perch, and carp fish muscles from West Pomerania River, Poland, were ranged 0.001–0.003 mg/kg. Dvořak et al. [60] recorded that the levels of Cd in the muscles of roach and chub (*Squalius cephalus*) in Dyje River was lower than 0.05 mg/kg. Juresa and Blanusa [61] observed the Cd level (0.002 μg/g) in hake fish muscles from the Adriatic Sea, Croatia. Łuczy´nska and Brucka-Jastrzębska [62] estimated Cd levels in various freshwater fish species from Poland. Cadmium maximum levels were recorded (3.6, 3.2, 2.8 and 3.0 μg/kg) from pike, bream, perch, and vendace, respectively. Zrelli et al. [63] estimated Cd levels in Fishery Products such as crustacean (0.09 ± 0.02 mg/kg) and crab sample *Portunus segnis* (3.45 mg/kg) from Tunisia.

### Health Risk Associated with the Intake of the Studied Species

#### Estimated Dietary Intake

Table 2 displayed EDI (μg/kg bw/w), EWI (μg/kg bw/w), PTWI%, MDI, and MWI of Cd in the muscles of *C. gariepinus*, *S. galilaeus*, *T. zillii*, O*. niloticus*, *and O. aureus* from Manzalah Lake that children, teenagers, and adults should intake*.*

**Table 2:** Health risk associated with the consumption of the investigated species from Manzalah Lake, Egypt

Intake	*C. gariepinus *			*S. galilaeus*			*T. zillii*			*O. niloticus*			*O. aureus*		
	Child	Teen	Adult	Child	Teen	Adult	Child	Teen	Adult	Child	Teen	Adult	Child	Teen	Adult
EDI	5.83	2.189	1.25	5.65	2.12	1.21	5.58	2.12	1.19	5.47	2.05	1.17	4.95	1.85	1.06
EWI	40.81*	15.32*	8.75*	39.55*	14.84*	8.47*	39.06*	14.84*	8.33*	38.29*	14.35*	8.19*	34.65*	12.95*	7.42*
PTWI	7.0	7.0	7.0	7.0	7.0	7.0	7.0	7.0	7.0	7.0	7.0	7.0	7.0	7.0	7.0
PTWI%	583	218.85	125	565	212	121	558	212	119	547	205	117	495	185	106
MDI	10.66	28.43	49.76	11.00	29.33	51.34	11.15	29.73	52.04	11.36	30.30	53.03	12.55	33.47	58.57
MWI	74.64	199.05	348.34	205.37	205.37	359.41	78.06	208.17	364.31	79.54	212.12	371.21	87.86	234.30	410.04

^*^above PTWI according to FAO/WHO, [30]

**Table 3:** Target hazard quotient (THQ) and target cancer risk (TCR) for Cd in five fish species from Manzalah Lake, Egypt

Fish	Non-carcinogenic risk THQ		Carcinogenic risk CR		
	Cd (once a week)	Cd (7 times a week)	Cd (once a week)		Cd (7 times a week)
Child					
* C. gariepinus*	5.83	40.81	2.21 × 10^−3^	1.55 × 10^−2^	
* S. galilaeus*	5.65	39.55	2.14 × 10^−3^	1.50 × 10^−2^	
* T. zillii*	5.58	39.06	2.12 × 10^−3^	1.48 × 10^−2^	
* O. niloticus*	5.47	38.29	2.07 × 10^−3^	1.45 × 10^−2^	
* O. aureus*	4.95	34.65	1.88 × 10^−3^	1.31 × 10^−2^	
Teens					
* C. gariepinus*	2.18	15.32	8.2 × 10^−4^	5.82 × 10^−3^	
* S. galilaeus*	2.12	14.84	8 × 10^−4^	5.63 × 10^−3^	
* T. zillii*	2.12	14.84	8 × 10^−4^	5.63 × 10^−3^	
* O. niloticus*	2.05	14.35	7.7 × 10^−4^	5.45 × 10^−3^	
* O. aureus*	1.85	12.95	7 × 10^−4^	4.92 × 10^−3^	
Adult					
* C. gariepinus*	1.25	8.75	4.7 × 10^−4^	3.32 × 10^−3^	
* S. galilaeus*	1.21	8.47	4.59 × 10^−4^	3.21 × 10^−3^	
* T. zillii*	1.19	8.33	4.5 × 10^−4^	3.16 × 10^−3^	
* O. niloticus*	1.17	4.95	4.4 × 10^−4^	1.18 × 10^−3^	
* O. aureus*	1.06	7.42	4 × 10^−4^	2.81 × 10^−3^	

In this research, Table 2 revealed that the EDI or EWI and PTWI% of Cd were ranked in the order of children > teenagers > adults depending on the consumption of five fish species muscles. Also, this study showed that the EDI, EWI, and PTWI% of Cd was in the rank of C*. gariepinus* > *S. galilaeus* > *T. zillii* > O*. niloticus* > *O. aureus* by children, teenagers, and adults. The current results were much higher than that was reported by the PTWI value established by FAO/WHO for Cd residue in five fish species*.* So, all fish species pose a great risk to children, teenagers, and adults’ health with must advice on safe levels MDI and MWI of all fish species.

Table 2 of this study demonstrated that the MDI and MWI of muscles of five fish species proposed that the MDI or MWI values for Cd that children should consume no more than 10.66 g/day or 74.64 g/week *C. gariepineus* muscle, 11.00 g/day or 205.37 g/week *S. galilaeus* muscle, 11.15/g/day or 78.06 g/week *T. zillii* muscle, 11.36 g/day or 79.54 g/week O*. niloticus* muscle, and 12.55 g/day or 78.86 g/week *O. aureus* muscle. Alternatively, teenagers should consume no more than 28.43 g/day or 199.05 g/week *C. gariepineus* muscle, 29.33 g/day or 205.37 g/week *S. galilaeus* muscle, 29.73 g/day 208.17 g/week *T. zillii* muscle 30.30 g/day or 212.12 g/week O*. niloticus* muscle, and 33.47 g/day or 234.30 g/week *O. aureus* muscle. Finally, adults should consume less than 49.76 g/day or 348.34 g/week *C. gariepineus* muscle, 51.34 g/day or 359.41 g/week *S. galilaeus* muscle, 52.04 g/day or 364.31 g/week *T. zillii* muscle 53.03 g/day or 371.21 g/week O*. niloticus* muscle, and 58.57 g/day or 410 g/week *O. aureus* muscle.

In agreement with our results, fish collected from Lake Edku recorded Cd levels in tilapia fish species that showed unhealthy risk consumption for children, teenagers, and adults [46]. Adversely, a study by Alipour et al. [49] recorded that EWI due to fish consumption of *Rutilus rutilus* from Iran was far below PTWI for Cd. Our findings were in disagreement with those published by Zaza et al. [64], who discovered that Italian consumer estimated weekly intakes of Cd in fish and shellfish products did not above the PTWIs established by EFSA and JEFCA. Ali et al. [65] recorded that Cd from Challawa River Northwestern Nigeria had elevated EDI and THQ values suggested that fish, from the Challawa River, were polluted and posed health potential risks if consumed. Similarly, the Cd intake due to consumption of five fish species from Lake Burullus posed a risk on human health for children, teenagers, and adults that were greater than PTWI [54].

### Non–carcinogenic Risk and Carcinogenic Risk

#### Target Hazard Quotient (THQ) and Target Cancer Risk (TCR)

The current findings demonstrated the THQs of Cd exposure due to the consumption of five fish species a day or 7 days a week from Manzalah Lake in Table 3, where the THQs values of Cd for the C*. gariepinus* > *S. galilaeus* > *T. zillii* > O*. niloticus* > *O. aureus* exceeded the safe value of one indicating that the consumption of these species a day or a week should cause a health risk for humans.

The current results reported that the THQs values of Cd level in children > teens > adults. Moreover, the THQ value of C*. gariepinus* intake by a child showed the highest value (5.83 a day or 40.81 a week) while *O. aureus* intake by an adult showed the lowest value (1.06 a day or 7.42 a week).

Target Cancer Risk of Cd via ingestion of five fish species from Manzalah Lake is displayed in Table 2. TCR Cd levels for *C. gariepinus* > *S. galilaeus* > *T. zillii* > *O. niloticus* > *O. aureus* above the USEPA [33] allowed limits, implying that consuming these species on a daily or weekly basis poses a substantial danger to humans [33].

Target Cancer Risks of Cd in five freshwater fish species from Manzalah Lake, Egypt, shown in Table 2 reported that Cd examined the TCR level in children > teens > adults. *C. gariepinus* in children had the greatest level of CR (2.04 × 10^−3^ a day or 1.55 × 10^−2^), whereas *O. aureus* in adults had the lowest level of CR (4 × 10^−4^ or 2.81 × 10^−3^). As a result, the possible danger of cancer for consumers from Cd-contaminated fishes should not be ignored.

Orajiaka-Uchegbu et al. [66] observed that the THQs of Cd from Ndoni and Choba creeks in Rivers State, Nigeria, were smaller than one assumed that no harm with noncarcinogenic Cd intake via tilapia, crab, and snail ingestion. Emam et al. [67] recorded that THQs values of Cd in catfish and tilapia from Edku, Egypt, were fewer than the limiting value of one for children and adults, showing the absence of possible noncarcinogenic risks.

According to Sallam et al. [53] consuming tilapia, catfish, and flathead gray mullet from Manzala Lake, Egypt, may pose a cancer risk to consumers. Said et al. [68] recorded that the average HQ calculated values through Cd consumption were reported higher than the permissible limit of 1 for all fish samples from the Phander Valley, Northern Pakistan; the elevated HQ values which are higher than the limit set may pose a significant chronic risk to the susceptible community. Yi and Zhang [69] observed that HI values were higher than one for the community and fishermen, respectively, implying that consumption of fish from the Yangtze River, China, could pose a risk. Mielcarek et al. [55] recorded that THQ of Cd of Brown trout, common bream, European eel, pike-perch, and vendace from Poland were non-carcinogenic; adversely, a high CR value were observed that may cause cancer. Copat et al. [70] observed that THQ of Cd from Mediterranean Sea fish from Sicily was less than 1.0 for adults indicated non-carcinogenic risk. Orajiaka-Uchegbu et al. [66] recorded no cancer risk from tilapia, snail and crab ingestion from Ndoni and Choba creeks, Rivers State, Nigeria. Storelli [71] observed that the THQs of Cd of fish were 0.01–0.04 and indicated that health risk was non-significant. Mansouri et al. [72] recorded that THQ < 1 for Cd indicated that the intake limit of fish was acceptable in Anzali Wetland (Iran). Nii Korley Kortei et al. [73] estimated THQ of Cd from Ankobrah and Pra basins, and the values ranged from 0 to 0.08 mg/kg. THQ of Cd for children and adult were > 1 which indicated a risk effects. Zhang and Wang [74] recorded that THQ of Cd in marine wild fish from China indicated a non-carcinogenic health risk.

## Conclusion

The present research manifested that the average concentrations of Cd in five species from Manzalah Lake, Egypt, were recorded as follows: *C. gariepinus* > *S. galilaeus* > *T. zillii* > *O. niloticus* > *O. aureus* (μg/g wet weight) that were largely above the permissible level of FAO/WHO and EC. The EDI and EWI were much higher than that was reported by the PTWI value established by FAO/WHO for Cd residue in five fish species*.* The investigation’s findings revealed that the five fish species represent a great risk to children > teenagers > adults health with essential advise on safe MDI and MWI levels for every fish species. THQs values of Cd for the C*. gariepinus* > *S. galilaeus* > *T. zillii* > O*. niloticus* > *O. aureus* exceeded the safe value of one indicating that the consumption of these species a day or a week should cause a health risk for humans. *C. gariepinus* in children had the greatest level of TCR, whereas *O. aureus* in adults had the lowest level of TCR. As a result, the possible danger of cancer for consumers from Cd-contaminated fishes should not be ignored. This study proved that Lake Manzala was one of the most polluted lakes, which supported the right decision of the Egyptian government under presidential directives to redevelop it to restore its economic, strategic, and logistical importance.

## Data Availability

All data is available with corresponding author.
